# Influence of sex on the requirement for and outcomes following late postnatal corticosteroid treatment

**DOI:** 10.1007/s00431-023-04826-3

**Published:** 2023-01-24

**Authors:** Rebecca Lee, Emily Kostina, Theodore Dassios, Anne Greenough

**Affiliations:** 1 Department of Women and Children’s Health, School of Life Course Sciences, Faculty of Life Sciences and Medicine, King’s College London, London, United Kingdom; 2 Neonatal Intensive Care Centre, King’s College NHS Foundation Trust, London, United Kingdom; 3 Asthma UK Centre for Allergic Mechanisms in Asthma, London, United Kingdom; 4 NIHR Biomedical Research Centre based at Guy’s and St Thomas NHS Foundation Trust and King’s College London, London, United Kingdom

**Keywords:** Prematurely born infants, Postnatal corticosteroids, Mortality, BPD

## Abstract

There remains a disparity between the outcomes of male and female prematurely born infants. Our aim was to assess the influence of sex on the requirement for late (> 7 days) postnatal corticosteroid (PNS) treatment and the outcomes following treatment. A retrospective whole population study of infants born at less than 28 weeks of gestation in all neonatal units in England between 2014 and 2018. The impact of exposure to at least five consecutive days of dexamethasone or hydrocortisone on bronchopulmonary dysplasia (BPD) at 36 weeks corrected gestation and survival to discharge from neonatal care was determined. Ten thousand, six hundred and fifty-five infants survived to seven days. Male sex was associated with an increased incidence of BPD (OR 1.41, 95%CI 1.287–1.552, *p* < 0.001) and death (OR 1.227, 95%CI 1.123–1.452, *p* < 0.001). Two thousand, three hundred and forty-four infants (22%) received at least one course of PNS at a median of 23 (IQR 15–40) days after birth. Males (23.6%) were more likely to receive PNS than females (20.1%), *p* < 0.001 and receive repeated courses (mean 1.67 compared to a mean of 1.59 in the females), *p* = 0.027. Multivariate regression analysis identified no significant differences in the incidence of BPD or death between male and females who received PNS.

*  Conclusions*: Males and females had similar outcomes after receiving PNS, but a significantly greater proportion of males met the clinical threshold to receive PNS and were more likely to receive repeated courses which may expose them to a greater risk of adverse long-term outcomes.

**Table:** 

**What is Known:** *• There remains a difference in outcomes of male and female infants born prematurely.* *• Prematurely born male infants were more likely to receive postnatal corticosteroids and a greater number of courses but had similar outcomes compared to female infants.*	
**What is New:** *• Postnatal corticosteroids have long-term adverse effects. Such outcomes should be considered when weighing up the risk–benefit ratio of prescribing postnatal corticosteroids, particularly in very prematurely born male infants.*

**What is Known:**

*• There remains a difference in outcomes of male and female infants born prematurely.*

*• Prematurely born male infants were more likely to receive postnatal corticosteroids and a greater number of courses but had similar outcomes compared to female infants.*

**What is New:**

*• Postnatal corticosteroids have long-term adverse effects. Such outcomes should be considered when weighing up the risk–benefit ratio of prescribing postnatal corticosteroids, particularly in very prematurely born male infants.*

## Introduction

Despite many advances in neonatal intensive care resulting in improved outcomes for extremely prematurely born infants, males compared to females have higher incidences of severe respiratory morbidity [1, 2] and mortality [2, 3]. Genetic [4], hormonal [5, 6], and functional [7] differences between male and female prematurely born infants have all been postulated to explain the disparities observed. Sexual dimorphism to antenatal corticosteroids has previously been assessed in a whole population analysis which demonstrated that antenatal corticosteroids (ANS) had a greater beneficial effect in female compared to male infants as evidenced by a reduction in mortality before discharge [8]. Inflammation leading to abnormal lung development is understood to play an important role in the development of BPD [9], and corticosteroids have strong anti-inflammatory effects. Postnatally delivered corticosteroids have been studied as to their role in preventing and treating BPD [10, 11]. In a large European observation study, male sex was independently associated with an increased likelihood of receiving postnatal corticosteroids (PNS) [12]. Despite this, it has not been assessed whether sex is an influencing factor on the response to PNS in the largest meta-analyses to date [11, 13].

Our aims, therefore, were to report the frequency of postnatal steroid (PNS) use and describe the cohort of infants who received PNS in a five-year whole population analysis. In addition, we wished to determine if the outcomes of bronchopulmonary dysplasia (BPD) at 36 weeks of corrected gestational age and death before neonatal discharge in infants who received postnatal steroids differed between males and females.

## Material and methods

A retrospective analysis of a whole population cohort of infants born at less than 28 weeks of gestation and admitted to any neonatal network in England during a five year period, 1^st^ Jan 2014 to 31^st^ Dec 2018 was undertaken. Data were provided by the National Neonatal Research Database (NNRD), Imperial College London, U,K and approved by the National Research Ethics Service (10/H0803/151), Confidentiality Advisory Group of the Health Research Authority (8–05[f]/2010), and the Caldicott Guardians and Lead Clinicians of contributing hospitals.

Patient demographic data included gestational age at birth in weeks and days since the last menstrual period or where unknown from the first trimester ultrasound, standardized birth weight *z*-score and sex. Diagnosis of moderate–severe BPD at 36 weeks corrected [14], postmenstrual age, corrected gestational age, and survival to discharge home were the main outcome variables.

To prevent the inclusion of infants who received corticosteroids for other indications such as hypotension in the early days following birth, we only included infants who survived to postnatal day seven. An episode of PNS exposure was dichotomously coded if the infants received hydrocortisone or dexamethasone for at least five consecutive days.

### Outcomes

BPD was diagnosed if the infant was receiving mechanical ventilation (MV), non-invasive respiratory support (NIV), or supplementary oxygen at 36 weeks corrected postmenstrual age [14]. Infants who were discharged prior to 36 weeks PMA on home oxygen were also categorized as having BPD. Infants were categorized as surviving if they did not die before neonatal discharge.

### Analysis

The Kolmogorov–Smirnov test was used to assess the data for normality, and the data were shown to be non-normally distributed. Differences, therefore, were assessed for statistical significance using the chi-squared test or Mann–Whitney *U* test. Multivariate logistic regression was performed to identify factors independently associated with the outcomes of interest for the effects of PNS exposure between male and female infants.

The statistical data analyses were performed using IBM SPSS software, version 27.

## Results

There were 11,855 infants in the full cohort of infants born prior to 28 weeks of gestation. Two thousand, three hundred and ninety-eight infants (20.2%) died before neonatal discharge, at a median of 6.95 days (IQR 1.33–23.51); 1200 of those infants died prior to seven days after birth.

Of the 10,655 infants who survived beyond seven days, 54.9% were male, with a median gestational age of 26.0 (IQR 24.86–27.14) weeks, birth weight of 809 (IQR 670–960) grams, and BW *z*-score of −0.385 (IQR −0.883–0.068) (Table 1). Eighty-seven percent were receiving respiratory support: mechanical ventilation (MV), non-invasive ventilatory support (NIV), or supplementary oxygen (O_2_) at day 28, with a median duration of 12 (IQR 4–28) days of MV.

**Table 1:** Full cohort demographic description, and comparison by sex in infants who survived to 7 days, *n* = 10,655

**Full cohort description**		**Comparison by sex**		
	***n* = 10,665**	**Males *n* = 5707**	**Females *n* = 4948**	***P* value**
**Gestational age**	26.14 (25.0–27.14)	26.28 (25.0–27.14)	26.14 (25.0–27.14)	0.036
**Gender (male)**	5707 (53.6%)	n/a	n/a	n/a
**Birth weight (grams)**	833.0 (686–970)	850 (710–1000)	790 (660–932)	< 0.001
**Birth weight (*z* score)**	−0.365 (−0.862–0.081)	−0.39 (−0.89–0.07)	−0.36 (−0.84–0.08)	0.007
**Antenatal steroids**	9537 (89.5%)	5127 (98.8%)	4410 (89.1%)	0.487
**Postnatal steroids**	2344 (22%)	1348 (23.6%)	996 (20.1)	< 0.001
**Surfactant given**	8878 (88%)	5103 (89.4%)	4380 (88.6%)	0.644
**Discharge weight (grams)**	2585 (2120–3175)	2670.0 (2170–3265)	2500.0 (2076–3045)	< 0.001
**Discharge weight (*z* score)**	−1.50 (−2.25– −0.81)	−1.508 (−2.223– −0.807)	−1.502 (−2.26– −0.824)	0.364
**Died before discharge**	1198 (11.2%)	710 (12.3%)	496 (10%)	< 0.001
**PMA discharge**	39.1 (36.89–41.8)	39.23 (36.91–41.95)	38.91 (36.86–41.62)	0.007
**Mechanical respiration (days)**	12 (4–28)	13 (4–30)	10 (3–27)	< 0.001
**Respiratory support at 28 days**	9311 (87.4%)	4990 (87.4%)	4321 (87.3%)	< 0.001
**Surgery for NEC**	1655 (15.5%)	954 (16.7%)	701 (14.2%)	< 0.001
**TPN (days)**	16 (11–27)	16 (11–27)	16 (11–26)	0.007
**IVH grade 3 or greater**	1512 (14.2%)	912 (16%)	600 (12.2%)	< 0.001
**ROP treatment**	1380 (13%)	770 (13.5%)	610 (12.3%)	0.064
**Cystic PVL**	442 (4.1%)	246 (4.3%)	220 (4.4%)	0.733
**Further analysis only including infants surviving to 36 weeks, *n* = 9614**				
		**Males *n* = 5321**	**Females *n* = 4677**	
**BPD**	6619 (68.8%)	3664 (71.8%)	2955 (65.5%)	< 0.001
**Home on supplementary oxygen**	3495 (36.3%)	1929 (36.2%)	1566 (33.5%)	< 0.001

Data demonstrated as median IQR or *n* (%)

*PMA *post menstrual age, *NEC *necrotizing enterocolitis, *TPN *total parenteral nutrition, *IVH *intraventricular hemorrhage, *ROP *retinopathy or prematurity, *PVL *periventricular leukomalacia, *BPD *bronchopulmonary dysplasia

Male and female infants were born at similar gestational ages; males 26.0 (IQR 24.68–27.14) weeks versus females 26.0 (IQR 24.86–27.0) weeks, *p* = 0.226, although male infants were born at a lower birth weight *z*-score, −0.382 (IQR −0.909– −0.062) than females, −0.366 (IQR −0.848–0.075), *p* = 0.008. There was no significant difference in exposure to antenatal corticosteroids, males 88.3% versus females 87.9%, *p* = 0.371.

Among infants who survived beyond postnatal day seven, male infants received more days 13 (IQR 4–30) of MV compared to females, 10 (IQR 3–27) days, *p* < 0.001 and were more likely to be receiving respiratory support at 28 days, *p* < 0.001. A greater proportion of males were discharged home in supplementary oxygen, 36.2% compared to 33.5% of females, *p* < 0.001. In a greater proportion of male infants, 71.8% were diagnosed as having BPD than females—65.5%, OR 1.413 (95% CI 1.287–1.552), *p* < 0.001. Twenty-two percent of male infants died before discharge compared to 18.5% of females, OR 1.277 (95% CI 1.123–1.452), *p* < 0.001.

In the cohort of infants that survived to seven days, 2344 infants (22%) received PNS. The mean number of courses received was 1.64 (SD ± 1.063) at a median postnatal age of 23 (IQR 15–40) days. Infants who received PNS were of a lower gestational age 25.0 (IQR 24.14–26.14) weeks versus 26.42 (IQR 25.42–27.28) weeks, *p* < 0.001 and had a lower BW *z*-score −0.558 (IQR −1.06– −0.07) vs. −0.32 (IQR −0.81–0.11), *p* < 0.001. A lower proportion was exposed to ANS; 88% versus 90%, *p* = 0.011, OR 1.2 (95%CI 1.023–1.407), *p* = 0.025.

Infants exposed to PNS had more mechanical ventilation days 35.0 (IQR 24–51) compared to those not exposed, 8.0 days (IQR 3–18), *p* < 0.001. They were more likely to be receiving respiratory support at day 28, 93.8% versus 86.5%, *p* < 0.001 and be diagnosed with BPD 91.7% versus 62.6%, OR 3.676 (95% CI 3.098–4.362), *p* < 0.001.

A greater proportion of infants exposed to PNS died before neonatal discharge 16.6% versus 9.7%. When considering, however, the influencing factors of GA and BW *z*-score, antenatal steroid exposure, and male sex, postnatal steroid exposure was associated with a reduction in the likelihood of death before discharge in multivariable logistic regression analysis, OR 0.855 (95% CI 0.740–0.989), *p* = 0.035.

A greater proportion of males (23.6%) than females (20.1%) received at least one course of PNS, OR 1.279 (95%CI 1.157–1.414), *p* < 0.001. Among those who did not receive PNS, the male infants were of higher gestational age (*p* < 0.001) and greater birth weight (*p* < 0.001) (Table 2). There were no significant differences in gestational age between male and female infants who received PNS (Table 3). The incidence of BPD was 91.9% in males and 91.5% in females who received at least one course of PNS, OR 1.003 (95% CI 0.727–1.384), *p* = 0.984. Male infants were more likely to require repeated courses of PNS, mean 1.67 (SD ± 1.099) compared to females, mean 1.59 (SD ± 1.010), *p* = 0.027. Both sexes received their first dose of PNS at a similar postnatal age: males 28.71 (IQR 27–31) days, females, 23 days (IQR 15–41), *p* = 0.679 (Table 4).

**Table 2:** Comparison between male and female infants (who survived to 7 days), who did not receive PNS

	**Male *n* = 4359**	**Female *n* = 3952**	***P* value**
**Gestational age**	26.57 (25.42–27.28)	26.42 (25.28–27.28)	< 0.001
**Birth weight (grams)**	900 (760–1035)	828.5 (700–960)	< 0.001
**Birth weight (*z* score)**	−0.332 (−0.81–0.11)	−0.318 (−0.80–0.118)	0.403
**Antenatal steroids**	3946 (90.5%)	3531 (89.3%)	0.199
**Surfactant given**	3820 (87.6%)	3441 (87.1%)	0.933
**Died before NNU discharge**	465 (10.7%)	343 (8.7%)	0.003
**Discharge weight (grams)**	2576 (2140–3100)	2425 (2050–2888.5)	< 0.001
**Discharge weight (*z* score)**	−1.45 (−2.14– −0.782)	−1.46 (−2.21– −0.819)	0.035
**PMA discharge**	38.67 (36.67–40.83)	38.46 (36.65–40.63)	0.204
**Mechanical respiration (days)**	9.0 (3–20)	7.0 (2–17)	< 0.001
**Respiratory support at 28 days**	3726 (85.5%)	3387 (85.7%)	< 0.001
**Surgery for NEC**	650 (14.9%)	504 (12.8%)	0.004
**TPN (days)**	15.0 (10–24)	14.0 (10–23)	0.215
**IVH grade 3 or greater**	636 (14.6%)	442 (11.2%)	< 0.001
**ROP treatment**	342 (7.8%)	318 (8%)	0.784
**Cystic PVL**	164 (3.8%)	129 (3.3%)	0.341
**Including only infants surviving to 36 weeks, *n* = 9614. Not receiving PNS *n* = 7545**			
	**Male *n* = 3919**	**Female *n* = 3628**	***P* value**
**BPD**	2577 (65.8%)	2146 (59.2%)	< 0.001
**Home on O**_**2**_	1347	1095	< 0.001

Data are demonstrated as *n* (%) or median (IQR)

*PMA *post menstrual age, *NEC *necrotizing enterocolitis, *TPN *total parenteral nutrition, *IVH *intraventricular hemorrhage, *ROP *retinopathy or prematurity, *PVL *periventricular leukomalacia, *BPD *bronchopulmonary dysplasia

**Table 3:** Comparison between male and female infants (who survived to 7 days), who received PNS

	**Male *n* = 1348**	**Female *n* = 996**	***P* value**
**Gestational age**	25.0 (24.14–26.14)	24.85(24.14–26.14)	0.078
**Birth weight (grams)**	721 (630–830)	656.5 (585–760)	< 0.001
**Birth weight (*z* score)**	−0.606 (−1.08– −0.08)	−0.489 (−1.03– −0.06)	0.078
**Antenatal steroids**	1181 (87.6%)	879 (88.3%)	0.706
**Surfactant given**	1283 (95.3%)	939 (94.4%)	0.932
**Died before NNU discharge**	237 (17.6%)	151 (15.2%)	0.119
**Discharge weight (grams)**	3130 (2430–3837)	2980 (2299–3631.25)	0.002
**Discharge weight (*z* score)**	−1.70 (−2.59– −0.883)	−1.63 (−2.48– −0.85)	0.172
**PMA discharge**	42.13 (39.32–45.55)	41.93 (39.01–45.42)	0.392
**Mechanical respiration (days)**	36.0 (24–51)	35.0 (23–50.75)	0.156
**Respiratory support at 28 days**	1264 (93.8%)	934 (93.8%)	0.868
**Surgery for NEC**	304 (22.6%)	197 (19.8%)	0.105
**TPN (days)**	25.0 (15–41)	24.0 (15–38)	0.174
**IVH grade 3, or greater**	276 (20.5%)	158 (15.9%)	0.018
**ROP treatment**	428 (31.8%)	292 (29.3.8%)	0.207
**Cystic PVL**	83 (6.2%)	66 (6.6%)	0.859
**Including only infants surviving to 36 weeks, *n* = 9614. Receiving PNS *n* = 2067**			
	**Male *n* = 1183**	**Female *n* = 884**	***P* value**
**BPD**	1087 (91.9%)	809 (91.5%)	0.763
**Home on O**_**2**_	582	471	0.165

Data are demonstrated as *n* (%) or median (IQR)

*PMA *post menstrual age, *NEC *necrotising enterocolitis, *TPN *total parenteral nutrition, *IVH *intraventricular hemorrhage, *ROP *retinopathy or prematurity, *PVL *periventricular leukomalacia, *BPD *bronchopulmonary dysplasia

**Table 4:** PNS administration in infants surviving to seven days, *n* = 2344

**Number of PNS courses**		1.64 mean (SD ± 1.063)	
**First dose (day)**		23.0 (15–40)	
**First dose PMA**		28.71 (27.00–31.14)	
**Analysis by sex:**			
	**Male *n* = 1183**	**Female *n* = 884**	***P* value**
**Number of PNS courses**	1.67 mean (SD ± 1.099)	1.59 mean (SD ± 1.010)	0.027
**First dose (day)**	28.71 (27–31)	23 (15–41)	0.679
**First dose PMA**	28.71 (27.00–31.00)	28.57 (26.85–31.14)	0.462

Data are demonstrated as *n* (%) or median (IQR), unless otherwise stated

*PMA* post menstrual age

A greater number of courses of PNS received was independently associated with an increased the likelihood of BPD at 36 weeks corrected gestational age, OR 1.822 (95% CI 1.411–2.352), *p* < 0.001.

There were no significant associations between male sex, OR 1.193 (95% CI 0.952–1.495), *p* = 0.125 or the number of PNS courses received, OR 1.001 (95% CI 0.903–1.110), *p* = 0.980, and the outcome of death before discharge in infants who received PNS.

## Discussion

We have demonstrated that in this whole population analysis of extremely prematurely born infants more than 20% received a course of PNS during their admission. Among the infants treated with PNS, however, we have found no evidence of a sexual dimorphism in BPD or death before neonatal discharge, but a greater proportion of male infants met the clinical threshold to necessitate treatment with PNS.

Sex was not assessed as an influencing factor in the most recent meta-analyses of PNS studies [11, 13]. On examination of the included studies in the Cochrane review [11], two studies reported a pre-planned subgroup analysis of the response to PNS exposure by sex. Kari et al. identified that the beneficial effect to dexamethasone which started at day 10 in infants of birthweight less than 1500 g was only seen in female infants [15]. The STOP-BPD study, however, reported that there were no significant differences between sexes with regard to outcomes. That study, however, found no beneficial effect of postnatal hydrocortisone compared to placebo overall [16].

Male infants who survived to day seven of life were more likely to receive a greater number of courses of PNS compared to female infants. There is a paucity of data on the effectiveness and safety of repeated courses of PNS; however, worse neurodevelopmental outcomes have been reported with increasing cumulative doses of PNS in longitudinal studies [17, 18]. Furthermore, a longer term study has identified a greater reduction in lung function in school aged children associated with larger cumulative doses of postnatal dexamethasone in infants born at less than 29 weeks of gestation [19]. It has recently been reported that 19 to 39% of PNS-treated infants received repeated courses of PNS with varying effectiveness in facilitating a step down of respiratory support in 38–93% of infants [20–22].

A strength of this study is the use of a national dataset that allowed us to create an overview of premature infants born in the UK between 2014 and 2018. There are some limitations to the study. The type of corticosteroid administered, nor the dosage or duration of treatment each infant received could not be identified. The study period of included infants, born between 2014 and 2018, however, encompassed the published recommendations from the American Academy of Paediatrics [23] and European Consensus Guidelines on the management of RDS [24, 25] that advised “short courses of low-dose dexamethasone to be considered for individual infants at high risk of, or with, severe chronic lung disease” after “one to two weeks of age.” Therefore, a degree of homogeneity with the use of PNS nationally during that time period would be expected.

In conclusion, in this whole population analysis of extremely premature infants, no significant differences in clinical outcomes in response to treatment with postnatal corticosteroids were identified between male and female infants. A greater proportion of male infants, however, received postnatal corticosteroids and had a greater number of courses exposing them to a higher risk of subsequent long-term adverse outcomes. A prospective study is required to determine if indeed a greater proportion of males compared to females have long-term adverse outcomes.

The views expressed are those of the authors and not necessarily those of the NHS, the NIHR or the Department of Health.

## Data Availability

Data will be shared on reasonable request.

## Abbreviations

ANS: Antenatal corticosteroids
BPD: Bronchopulmonary dysplasia
MV: Mechanical ventilation
NIV: Non-invasive respiratory support
PNS: Postnatal corticosteroids
